# Supplementary material for: SEANN: A domain-informed neural network for epidemiological insights
Source: PLoS One. 2025 Dec 16;20(12):e0338400. doi: 10.1371/journal.pone.0338400 (PMC12707678; doi:10.1371/journal.pone.0338400)
Supplement: Attachment — Submitted filename: SEANNanswerReviewers.pdf [file pone.0338400.s002.pdf]

SEANN: A Domain-Informed Neural Network for Epidemiological Insight (PLOS ONE)
Point by point response to reviewers

Reviewer #1:

General Comments:

The manuscript presents a novel and relevant approach by introducing SEANN, a domain-informed neural network model that leverages pooled effect sizes (PES) to enhance prediction and interpretation in epidemiological datasets. The work is timely and contributes to the growing intersection of machine learning and health sciences, especially in low-data settings. The manuscript is written in good English; however, it requires minor revisions before it can be accepted for publication.

Specific Comments:

1. The manuscript, while clearly written, lacks consistent formatting throughout. Please ensure that headings, equations, and paragraphs follow the journal's style guide. Several in-line equations are not properly spaced and disrupt the flow of reading.

Response:

Thank you for this observation. We have carefully reviewed the manuscript to ensure consistent formatting in accordance with *PLOS ONE*'s style guide. Specifically:

- All section and subsection headings have been adjusted to follow the journal's formatting conventions.
- Inline equations have been revised to include appropriate spacing to avoid disrupting the text flow.

2. Authors should check for the typographical errors such as in the Introduction section: "Odd Rations (ORs)" should be revised to "Odds Ratios (ORs)."

Response:

Thank you for pointing this out. We have corrected the typographical error in the Introduction section, replacing "Odd Rations (ORs)" with "Odds Ratios (ORs)".

We have also performed a thorough proofreading of the manuscript to identify and correct any other remaining typographical mistakes.

3. Authors should check for citing relevant citations in the text for e.g. On Page 5, the following line is missing a relevant citation:
"For a vector V of SRCs derived from the literature, with vi ∈ V, the ith element of V, the training loss term Lmeta is defined as..." Cite appropriate literature to support the use of the custom loss formulation.

Response:
We thank the reviewer for this suggestion. The loss formulations introduced before Eq. 2, Eq. 3, and Eq. 4 are novel and have not been presented in previous work. We have therefore clarified in the manuscript that these formulations are new, while citations to related works that inspire the general strategy of incorporating domain knowledge through additional loss terms [4, 18, 19] are provided in the Related Work section.

4. While the analysis appears comprehensive, the discussion lacks sufficient depth. It is recommended that the authors elaborate more on how their results compare with prior methods and explain the implications of improved generalizability in real-world epidemiological studies.

Response:
We thank the reviewer for this constructive feedback. In the revised manuscript, we have expanded the Discussion section to provide a comparison with prior methods and to better articulate the real-world implications of our findings.
The relevant additions (lines 378 - 402) are :

“[...] Our findings complement existing informed-ML approaches that encode domain knowledge as structural or mechanistic soft constraints (e.g., physics-guided networks or monotonic/range constraints implemented via loss regularization). In contrast, while prior work has injected effect-size estimates into simpler models such as naïve Bayes (e.g., CALIBRA), to our knowledge no method has integrated pooled effect sizes directly into neural network training, despite their prominence in epidemiological research.

Our experimental protocol demonstrates that, compared with an agnostic DNNs with similar architecture, our approach offers two main benefits. First, improved generalization to unseen data: when PESs encode relevant information that is weak or absent in the observed data, SEANN’s predictive performance degrades more slowly under noise and missingness, yielding higher out-of-sample accuracy. Second, improved alignment of extracted relationships with external knowledge: SEANN preserves epidemiologically plausible dose–response patterns for both informed and uninformed variables and can be used to better disentangle individual input-output relationships in the presence of collinearity. For real-world epidemiological studies, these benefits imply more reliable risk predictions across different cohorts, populations or sites and more stable, policy-relevant, effect estimates despite measurement error, limited sample sizes, and partial confounding.”

Reviewer #2:

General Comments:

This manuscript introduces a novel approach, SEANN (Summary Effects Adapted Neural Network), which integrates pooled effect sizes (PESs) such as Odds ratios, Risk ratios, and standardized regression coefficients directly into deep neural network training. This is a unique and timely contribution to informed machine learning, particularly relevant to fields like

epidemiology, where data is often limited or noisy. SEANN helps improve both predictive performance and interpretability by embedding prior scientific knowledge into model training.

Specific Comments:

1. The methodology is technically solid and clearly presented. The paper explains how PESs are embedded via custom loss functions and how weighting is derived based on confidence (sample size). The treatment of three common types of PESs is thorough. However, while the math is sound, the use of complex symbols and notations (Example: perturbation steps, arrow symbols) might challenge readers outside machine learning. Adding a simpler example with numeric inputs could help clarify the non-expert audiences.

Response:
We thank the reviewer for highlighting both the technical clarity and the potential accessibility issue for non-expert readers.
To address this concern, we have added new illustrative numeric examples in an **annex** that walk through the computation of the PES-informed loss using synthetic values. This example shows:

- A sample pooled effect size for each form (e.g., SRC = 2.0, OR = 1.5, RR = 1.2),
- How the input matrix is perturbed, and
- How the custom loss term is computed.

2. The experiments are well-structured and simulate realistic epidemiological challenges noise, missing data, and confounding. The use of synthetic data is reasonable for isolating the effects of the method. SEANN consistently outperforms standard DNNs in terms of both prediction and interpretability, especially when data is imperfect. The use of the SHAP metric to assess alignment with true relationships is particularly valuable. However, the study would benefit from including comparisons with other informed machine learning models, like PGNN or DANN, or at least discussing their relative merits.

Response:
We appreciate the reviewer’s positive evaluation of our experimental design and the use of SHAP for interpretability analysis.
In response to the suggestion, we have now expanded the *Related work* section (Page 2 of the revised manuscript) to discuss more clearly the merits of comparable existing methods incorporating domain knowledge into DNNs, as well as their differences with SEANN. Namely:

· **PGNNs (Physically Guided Neural Networks)** constrain predictions using physical models and are effective in domains with well-established governing equations such as hydrology or climate.

· **DANNs (Domain-Adapted Neural Networks)** incorporate domain-specific structural constraints such as monotonicity, known valid ranges, or approximation constraints.

We now clarify that none Sof those methods explicitly incorporates quantified prior statistical associations (i.e., effect size estimates: ORs, RRs, SRCs) directly into training which requires specific adaptations of the loss regularization that we provide in this work.

3. SEANN enhances the interpretability of model predictions, aligning well with real-world needs in health research. For example, it corrects misleading associations when variables are confounded or missing. While the figures and quantitative results are strong, some brief narrative explanations of what those corrections mean in practice, especially for health researchers, would make the work more engaging and accessible.

Response:

We thank the reviewer for this thoughtful suggestion. To better highlight the practical relevance of SEANN's interpretability gains, we have added paragraphs explaining what they mean in practice from a health research perspective for experiment 1 (lines 304 - 315) and experiment 2 (lines 332 - 342).

4. This is a strong and original piece of work. To improve it further, the authors should consider: Including a real-world dataset example.

Response:

We thank the reviewer for his positive feedback and suggestions.

We fully agree that applying SEANN to real-world data is important to demonstrate its utility beyond synthetic experiments. To this end, we would like to highlight that we have conducted and now published a real-world application of SEANN in the context of hypertension risk prediction, using data from a large-scale European birth cohort. This study was recently published in *Artificial Intelligence in Medicine*:

Guimbaud JB, …, Maître L, Cazabet R. An informed machine learning based environmental risk score for hypertension in European adults. Artif Intell Med. 2025 Jul;165:103139. doi: 10.1016/j.artmed.2025.103139. Epub 2025 Apr 22. PMID: 40311152.

This work was purely applicative and did not present SEANN in detail nor explore its theoretical benefits on simulated data.

Reviewer #3:

While the paper presents a creative idea — integrating domain knowledge in the form of pooled effect sizes into a neural network via a modified loss function — the manuscript ultimately lacks sufficient scientific rigor and practical relevance to warrant publication in PLOS ONE.

We thank the reviewer for their comment. While we believe that our approach has practical relevance (alignment with PES improves interpretability, trustworthiness, and may reduce spurious associations), we understand that perceived practical impact can vary by reader and use case. As PLOS ONE's editorial criteria emphasize rigor and transparency rather than

perceived impact or novelty, our primary goal has been to ensure that the work is technically sound, ethically conducted, and clearly reported.

My detailed concerns are as follows:

1. Lack of Real Data: The manuscript claims to improve epidemiological modeling, yet it contains no experiments on real-world epidemiological datasets. Synthetic toy examples (e.g., fish, mercury, stress, BMI) are insufficient to demonstrate the utility of the approach. Without validation on observational datasets from real health studies, the results cannot be trusted or generalized.

   Response:

   We appreciate the reviewer's concern regarding real-world validation, and we agree that applying novel methods to observational datasets is critical for assessing their practical relevance.

   We would like to clarify that the present manuscript is intended as a methodological contribution, with a focus on controlled benchmarking, ablation studies, and interpretability evaluation using synthetic but epidemiologically informed simulations. These settings allow us to isolate the effects of SEANN's architecture and loss formulation in ways that are difficult to achieve on observational data alone.

   That said, we have already validated SEANN on a real-world epidemiological dataset, as reported in a recent publication:

   Guimbaud JB, …, Maître L, Cazabet R. An informed machine learning based environmental risk score for hypertension in European adults. Artif Intell Med. 2025 Jul;165:103139. doi: 10.1016/j.artmed.2025.103139. Epub 2025 Apr 22. PMID: 40311152.

   This study applied SEANN to hypertension risk prediction using rich environmental and sociodemographic data from a European cohort. We demonstrate how SEANN improves performance and interpretability in a high-dimensional, partially confounded observational setting. However, again, this work was purely applicative and did not present SEANN in detail nor explore its theoretical benefits on simulated data.

   We have now cited and summarized this real-world application in the *Discussion* section (Page XX) of the current manuscript to better contextualize the present work as such:

   "Beyond these controlled synthetic experiments, SEANN has already been applied to real-world epidemiological data. In a recent study on hypertension risk in European adults, we integrated SEANN into the construction of environmental risk scores using observational cohort data (from the GCAT project) combining diverse environmental, sociodemographic, and clinical exposures. In this high-dimensional and partially confounded setting, SEANN improved the quality of captured relationships compared with agnostic neural networks, confirming its practical utility for epidemiological modeling. While that work was focused on the application and did not detail SEANN's methodological formulation, the present manuscript provides the complementary

theoretical and experimental foundations. Together, these contributions highlight SEANN's value both as a methodological innovation and as a practical tool for analyzing complex health data."

2. Overclaiming Practical Use: The term "epidemiological insights" in the title and abstract is misleading. No insights about disease risk, exposure, or population health are produced in this study. The method is demonstrated only in artificial settings, using pre-defined effects, which defeats the purpose of "insight" discovery.

Response:

We thank the reviewer for pointing out this potential ambiguity. Our intent with the term "epidemiological insight" was not to imply that new scientific knowledge about disease risk was uncovered in this manuscript, but rather to highlight how SEANN, by improving the alignment between model behavior and epidemiological knowledge, and by reducing spurious associations, can improve the quality of captured relationships (the insights) in epidemiological settings. Also, as stated in the previous comment, the benefits of our approach in epidemiological settings have now been validated on real observational data in this work [1]

To avoid any misunderstanding, we slightly revised the abstract to state more explicitly that the current manuscript focuses on evaluating methodological properties (predictive accuracy and attribution fidelity) under controlled experimental simulations, not on the discovery of new associations in real-world populations.
We hope this revision addresses the reviewer's concern.

3. Weak Baselines and Limited Evaluation: The authors compare SEANN only to an "agnostic DNN" — essentially a DNN without the PES-informed loss — and omit any comparison to:

- Classical epidemiological methods (e.g., regularized logistic regression with prior constraints),
- Knowledge-regularized models in causal inference,
- Other informed machine learning models in epidemiology (e.g., those incorporating DAGs or monotonic constraints).
- The absence of meaningful baselines diminishes the credibility of the claimed improvements.

Response:

We thank the reviewer for his comment. Our primary objective in this study was to isolate and evaluate the specific contribution of the PES-informed loss term. For this reason, we focused on comparing SEANN to an otherwise identical neural architecture (the agnostic DNN), in order to control for confounding design differences and attribute observed improvements specifically to the integration of PESs.

We acknowledge that comparisons to other modeling families could be of interest. However, our choice of baselines was motivated by the following considerations:

- Classical epidemiological models (e.g., logistic regression, regularized regression) have already been extensively compared to neural networks in the literature. In most cases, neural networks perform similarly or better on predictive tasks, but they do not naturally incorporate domain knowledge unless explicitly modified. Our aim here was not to revisit this comparison, but to demonstrate how PES integration alters a neural network's behavior relative to its agnostic counterpart.
- Knowledge-regularized approaches in causal inference or informed ML (e.g., DAG-based models, monotonic constraints) rely on fundamentally different forms of prior knowledge (graphical structure, monotonicity). These are not directly comparable to SEANN, which is designed to integrate statistical effect-size estimates—a type of prior that is abundant in epidemiology but not inherently represented in DAGs or monotonic constraints. Thus, a quantitative, experimental direct comparison between those methods and SEANN does not seem possible. We did extend the qualitative comparison with those methods, by detailing why they cannot solve the same problems as SEANN.

To our knowledge, the closest published approach is CALIBRA (Neri et al. 2022), which integrates PESs into a naive Bayes model. While this shows the feasibility of PES integration in a simpler setting, our focus here is to extend such integration to neural networks and demonstrate its implications for predictive stability and interpretability.

4. Unconvincing Justification of PES Integration: The proposed method modifies the loss function using hand-selected perturbation values (e.g., h=1 or h=1/vi), but these choices are not theoretically or empirically justified. There is also no sensitivity analysis regarding how these perturbations affect training stability or generalization. Furthermore, integrating PES values — which are often heterogeneous and context-dependent — as “hard-coded” truth in synthetic experiments does not reflect how they behave in actual epidemiological studies.
Response:

We appreciate this critical and thoughtful feedback. This comment raises several important concerns regarding both the formulation and evaluation of the PES-based integration strategy. We address them below in turn:

1. On the choice of perturbation values (h = 1 or h = 1/vi):

These values were selected to balance numerical stability and interpretability. We agree that the justification in the original manuscript was limited. To address this, we have now added a dedicated paragraph in the Methods section (Page 3) explaining the rationale:

- For SRCs, we set *h = 1* which makes the induced changes in predicted outcomes directly interpretable in terms of the PES magnitude. This is consistent with standard

practice in gradient-based effect estimation, where unit changes are often used to probe marginal effects.

- For ORs, the Lmeta loss is expressed as :

$$\mathcal{L}_{\text{meta}}(\mathbf{X}, \theta, v_i, h_i) = \frac{1}{n} \sum_{k=1}^{n} \left(\frac{e^{v_i h_i}\, p_\theta(\mathbf{X}_k^{x_i - h_i})}{e^{v_i h_i}\, p_\theta(\mathbf{X}_k^{x_i - h_i}) - p_\theta(\mathbf{X}_k^{x_i - h_i}) + 1} - p_\theta(\mathbf{X}_k) \right)^2$$

We set $h_i = \frac{1}{v_i}$, so that $e^{v_i h_i} = e$ in order to avoid exploding gradients and improve numerical stability during training. Scaling the perturbation inversely with effect size ensures that any PES values can be integrated without destabilizing the optimization.

These choices are intended as reasonable defaults rather than fixed requirements. Alternative perturbation schemes could be explored, for instance with a sensitivity analysis, without changing the general principle of SEANN.

2. On PES heterogeneity and realism in synthetic data:

Our goal was not to treat PES values as "hard-coded truths", but rather as structured external information that can guide model training. This is conceptually similar to how pooled effect size estimates are used in the literature, for instance to set priors in Bayesian regression (e.g., CALIBRA). In SEANN, PES values act as targets the model is encouraged to align with, rather than constraints it must exactly satisfy. The regularization term thus serves as a soft prior rather than a fixed truth, leaving the model free to adapt when observed data diverge from external estimates.

The use of synthetic data in our experiments was intended to isolate the contribution of this mechanism under controlled conditions. As noted in our response to Reviewer 2, SEANN has already been validated in a real-world setting [1], which demonstrates its practical applicability when faced with heterogeneous and context dependent PES estimates in a real epidemiological setting.

5. Limited Contribution to Machine Learning or Epidemiology: While the paper claims novelty in the integration of PESs into neural networks, the broader field of informed ML has explored similar loss regularization strategies with more robust theory and applications. This work does not meaningfully advance the methodological or epidemiological literature beyond existing approaches.

Response:

We respectfully disagree with the reviewer's assessment and would like to clarify the scope and originality of our contribution.

While it is true that the informed machine learning field has explored various regularization strategies, including physics-informed and domain-informed losses, to the best of our knowledge SEANN is the first model that systematically integrates quantitative *pooled effect sizes* (PESs) — such as odds ratios, risk ratios, and standardized regression coefficients — into deep neural network training as a form of meta-analytic guidance.

What distinguishes SEANN is:

· A conceptual framework for accommodating common epidemiological effect measures into neural network training, which has not been addressed in prior IML work.

· A customized loss formulation tailored to the mathematical nature of each PES type. Existing informed ML methods (e.g., physics-informed losses, monotonic constraints, DAG-based priors) are not designed to accommodate these statistical effect sizes, and adapting them, when possible, would require substantial methodological innovation.

· The added benefits of PESs incorporation specifically, which are one of the most trusted and abundant forms of prior knowledge in population health research, and their integration enables improved generalizability, reduced susceptibility to spurious associations, and better disentanglement of individual exposure effects in noisy, confounded data settings.

We do not claim that SEANN is a breakthrough for the entire ML community, but we argue that it fills a specific but important gap in epidemiological studies aiming to explore exposure effects on health using non-parametric modeling.
We believe this justifies its value as a methodological contribution tailored to the needs of health data science.

6. Figures and Tables Are Minimal and Not Informative Enough: The visualizations do not adequately explain how SEANN outperforms baselines. Key graphs (e.g., SHAP plots) are not contextualized with respect to meaningful health variables, and only small performance differences are observed in some metrics.

We thank the reviewer for the feedback. To improve clarity and informativeness, we have reformulated the figure captions for (Fig. 1–3 and Table 1) to:

1. State precisely what each axis/metric represents
2. Clarify that lower is better for ΔShap
3. Connect the visuals to the epidemiological narrative.

Regarding the small differences observed "in some metrics" we assume the reviewer is referring to experiment 1 when noise distortion or data missingness is small to null. In those regimes, both models operate near the ground-truth, so R^2/ROC-AUC differences are expected to be small. When the amount of distortion increases, SEANN separates more clearly from the agnostic DNN.
In experiments 2 and 3 the differences observed with the used metric (ΔShap) are significant.

References:

1. Guimbaud, J.-B. *et al.* An informed machine learning based environmental risk score for hypertension in European adults. *Artif. Intell. Med.* **165**, 103139 (2025).
